# Breast Carcinogenesis during Pregnancy: Molecular Mechanisms, Maternal and Fetal Adverse Outcomes

**DOI:** 10.3390/biology12030408

**Published:** 2023-03-06

**Authors:** Georgia Margioula-Siarkou, Chrysoula Margioula-Siarkou, Stamatios Petousis, Eleftherios Vavoulidis, Kosmas Margaritis, Aristarchos Almperis, Costas Haitoglou, George Mavromatidis, Konstantinos Dinas

**Affiliations:** 1 2nd Department of Obstetrics and Gynaecology, School of Medicine, Faculty of Health Sciences, Aristotle University of Thessaloniki, 54124 Thessaloniki, Greece; 2 2nd Department of Paediatrics, School of Medicine, Faculty of Health Sciences, Aristotle University of Thessaloniki, 54124 Thessaloniki, Greece; 3 Laboratory of Biochemistry, School of Medicine, Faculty of Health Sciences, Aristotle University of Thessaloniki, 54124 Thessaloniki, Greece

**Keywords:** breast cancer, carcinogenesis, pregnancy, pregnancy-associated breast cancer, molecular mechanisms, adverse maternal outcomes, adverse fetal outcomes

## Abstract

**Simple Summary:**

Pregnancy-associated breast cancer (PABC) is mainly defined as breast cancer diagnosed during the gestational period or in the first year after birth. Significant differences are detected between PABC and non-pregnancy-associated breast cancer, in terms of epidemiology, clinical manifestations, diagnostic and therapeutic management, and, most importantly, in biological behavior and pathophysiological basis. Hormonal and immune changes during pregnancy, breast involution and altered gene expression are recognized as potential contributors to the pathogenesis of PABC. There is considerable scientific interest in the prognosis of PABC, since various reported adverse maternal and fetal outcomes are induced by PABC, such as reduced maternal survival compared to non-pregnant patients with breast cancer, as well as obstetrical complications (predominately preterm delivery), fetal complications (mainly prematurity-induced neonatal diseases) and fetal malformations as a result of treatment administered during gestational period. Currently, there are no long-term adverse outcomes reported for children born from women with PABC who received treatment during pregnancy. The longitudinal observation of PABC survivors and their children may reveal new, currently undocumented short- and long-term complications and adverse outcomes.

**Abstract:**

Breast cancer is a common type of cancer diagnosed during pregnancy, with increasing incidence over the last years, as more women choose to delay childbearing. Compared to breast cancer in general population, pregnancy-associated breast cancer (PABC) is significantly different in its terms of epidemiology, diagnostic and therapeutic management, while it exhibits particularly aggressive behavior, deriving from its unique molecular and biological profile. Although not fully elucidated, the pathophysiological basis of PABC can be traced back to a combination of hormonal and immune changes during pregnancy, breast involution and altered gene expression. There is considerable controversy in the existing literature about the influence of PABC on pregnancy outcomes, regarding both short- and long-term effects on maternal and fetal/neonatal health. The majority of PABC patients have advanced-stage disease at initial diagnosis and face a significantly poorer prognosis, with decreased survival rates. The most commonly reported adverse obstetrical–fetal events are preterm delivery and prematurity-associated neonatal morbidity, while other neonatal treatment-associated complications might also occur, even when safe therapeutic options are applied during pregnancy. The objective of the present comprehensive review was to summarize current knowledge and up-to-date evidence about the pathophysiological, molecular and biological basis of PABC, as well as its association with adverse maternal, obstetrical, fetal and neonatal outcomes.

## 1. Introduction

Pregnancy-associated breast cancer (PABC) is mainly defined as breast cancer diagnosed during gestation or in the first year after birth, although the definition may vary with duration and length of postpartum period [1]. As research around this topic was evolving and potential pathophysiological connections between pregnancy, postpartum period and breast cancer development were explored, it was even proposed to define all breast cancer cases being diagnosed up to two years after birth as PABC [2]. Nowadays, it is gradually being accepted that, due to significant differences mainly in the diagnostic and therapeutic approach applied, breast cancer cases occurring during pregnancy and the postpartum period should be recognized as separate and distinct clinical entities. Thus, the term PABC tends to be gradually abandoned, and has been replaced by the terms “breast cancer in pregnancy (PrBC)” and “postpartum breast cancer (PPBC)” [1,3,4].

The incidence of PABC has been rapidly increasing from the beginning of the millennium, currently affecting approximately 17.4–39.9 per 100.000 deliveries annually, marking breast cancer as the second most common type of cancer diagnosed in pregnant women. Specifically, the incidence of PABC is significantly higher during the post-partum period (13.8–32.2 per 100.000 deliveries), compared to the gestational period prior to delivery (3.0 to 7.7 per 100.000 deliveries), a difference that can be explained by the fact that many masses discovered during pregnancy can be perceived as physiological changes and are likely to be initially ignored by the patient [5,6]. The increasing prevalence of PABC, which is more evident in developed countries and is expected to further rise in the following decades, is mostly attributed to delayed childbearing and advanced maternal ages at birth, since a great proportion of women choose to postpone their first delivery in order to pursue educational and professional opportunities [7]. 

Apart from its distinct epidemiology, PABC is drastically different from breast cancer in non-pregnant patients in terms of its clinical manifestation, and diagnostic and therapeutic management. The physiological changes of the breasts during pregnancy and lactation may conceal the presence of a mass in the breast or the axilla in pregnant women, and other symptoms such as breast pain, skin and nipple abnormalities, nipple retraction and discharge can be attributed to breastfeeding-related irritations and injuries, leading to a profound delay in the diagnosis of breast cancer, as well as a more locally advanced disease at time of diagnosis [8,9]. There are limitations in the use of diagnostic modalities to successfully identify breast cancer in women during pregnancy and lactation; the increased density of the breast in such patients may decrease the sensitivity of imaging methods (especially mammography), and other imaging tests necessary for accurate staging are avoided, due to the potentially harmful effects of radiation or contrast media to the fetus; even biopsies of suspicious masses are performed with caution when indicated, as they may be scarcely complicated by the formation of milk fistulas [10,11]. Regarding treatment, PABC requires the careful design of therapeutic strategies that can be substantially different from those applied to general population, as pregnancy poses significant restrictions to the use of some effective treatment options due to potential teratogenic effects to the fetus, especially systemic therapies such as radiation, chemotherapy, hormonal therapy and immunotherapy [12,13]. 

The differences between PABC and breast cancer in the general population are not limited to their epidemiology, clinical manifestations, and diagnostic and therapeutic management (Table 1). PABC also demonstrates a particularly aggressive biological behavior, including large a tumor size, nodal metastasis, high histologic grade, negative estrogen and progesterone status, and HER-2 overexpression [14,15]. The molecular nature behind these distinct characteristics of PABC is still not clarified and remains within a field of increasing research efforts, since it is evident that different biological, genetic and pathophysiological pathways are involved in the development of PABC. In addition, there is considerable scientific interest in the prognosis of PABC. There are various reported adverse maternal and fetal outcomes induced by PABC, such as reduced maternal survival compared to the general population, obstetrical complications, and fetal malformations as a result of applied treatment [16,17]. Therefore the longitudinal observation of PABC survivors and their children may reveal new, currently undocumented short- and long-term adverse outcomes. 

The objective of the present comprehensive review was to effectively summarize and present current knowledge and up-to-date evidence about the pathophysiological, molecular, and biological basis of PABC, as well as its association with short- and long-term adverse maternal, obstetrical, fetal and neonatal outcomes.

## 2. Pathophysiological, Biological and Molecular Basis of PABC

The protective effect of full-term pregnancy is a well-established concept, which has been extensively investigated and even has been proven and replicated in animal models, mostly rodents [18]. The completion of a full-term pregnancy at an early age, especially before 24 years of age, offers protection against breast cancer, leading to a reduction in lifetime risk by up to 50% [19]. The molecular and pathophysiological mechanisms responsible for this protective effect are not yet fully elucidated; it is suspected that a combination of procedures including lobular differentiation, changes in cell fate, and alterations in the parous stromal microenvironment during breast involution are involved [20]. The pregnancy-induced breast cancer protection theory further highlights the paradox of PABC, leading to the hypothesis that the pathophysiology, biological and molecular mechanisms of PABC should be substantially different, compared to the pathophysiological basis of non-pregnancy-associated breast cancer.

The completion of the first full-term pregnancy actually activates two potentially opposing effects on the risk of developing breast cancer, known as the dual effect of pregnancy, which can be roughly defined as pregnancy-induced activation, both of molecular mechanisms that promote cell proliferation and tumorigenesis, and of others that exert cancer-preventive properties. So, apart from invoking a lifetime reduction in risk of breast cancer development, pregnancy is also associated with a transient increase in breast cancer risk for up to 15 following years, during which all parous women, regardless of age, face a higher incidence of the disease, compared to nulliparous women. A delay in childbearing, especially after 35 years of age, further enhances this transient risk for subsequent breast cancer. As a result, an advanced maternal age at first birth is translated to an increase in the peak incidence of breast cancer in the immediate years postpartum, leading to a gradual steady increase in the prevalence of PABC [21,22,23,24].

Despite the fact that the pathophysiology of PABC remains unclear to a significant extent, several potential molecular mechanisms and pathways have been identified as being involved in the pathogenesis of PABC, probably having a synergistic combined effect in its development and progression [25,26]. First of all, the unique hormonal profile of pregnancy, characterized by increased levels of circulating estrogen, progesterone, and growth factors, especially IGF1, contributes to the enhancement of breast cell proliferation, and potentially triggers oncogenic transformation. More specifically, hormonal changes promote a particularly rapid progression of the tumor, from precancerous lesions and early stage disease into advanced stage and metastatic PABC, potentially by stimulating the proliferation of cells that have already been subjected to malignant transformation [21,25,27,28]. Additionally, the notable immune changes observed during pregnancy, including cellular immunosuppression, immune tolerance, and enhanced inflammatory responses triggered by breast involution, may also play an important role in the pathophysiology of PABC. The pregnancy-associated immune tolerance, which ensures that cells from the semi-allogenic fetus are able to escape immune control, also allows cancerous breast cells to overcome detection from the immune system and proliferate without being detected [25,27,29,30]. Finally, breast involution, normally observed after the gestational and lactation period, is recognized as a significant potential mechanism contributing to the development of breast cancer. During involution, the fully differentiated mammary tissue returns to its less mature, pregestational state. This complex transformation process includes apoptosis of the epithelial cells, extensive stromal remodeling, adipogenesis, and the initiation of various inflammatory reactions, sharing mutual characteristics with wound repairing and inflammatory microenvironments, which are known to exert pro-oncogenic properties [25,31,32,33,34].

Significant differences are also detected regarding gene expression patterns in breast tumors detected in patients with PABC and non-pregnancy-associated breast cancer [35]. PABC epithelial cells have additional copy numbers of DNA coding genes for morphogenesis, angiogenesis and metastases, as well as fewer copy numbers of DNA coding genes for tumor suppressors, cell adhesion, macromolecular complex assembly, and intra-cellular trafficking, changes that accumulatively lead to increased invasiveness and biological aggressiveness [25,27]. Epithelial cells of PABC demonstrate an enhanced expression of genes whose expression regulates immune responses, cell cycle regulation, metabolism, and aggressiveness, including estrogen- and progesterone-regulated genes associated with recurrence [36]. Genomic profiling via whole-genome sequencing has been proven to be very efficient in accurately identifying genes whose expression in PABC cells is significantly different. More specifically, PABC epithelial cells are characterized by an increased expression of various oncogenes (e.g., MYC, SRC, FOS), tumor suppressor genes (e.g., TP53, PTEN, CAV1), apoptosis regulators (e.g., PDCD4, BLC2, BIRC5), transcription regulators (e.g., JUN, KLF1, SP110), genes associated with DNA repair mechanisms (e.g., Sig20, BRCA1/2, FEN1), cell proliferation genes (e.g., AURKA, MKI67), and genes regulating the activation of immune response (e.g., PD1, PDL1) [37,38]. Furthermore, PABC is associated with a significantly higher frequency of non-silent mutations and mutations in the mucin gene family [39], as well as with an increased prevalence of germline mutations in genes that are known to predispose one to cancer (especially BRCA1 and CHEK2) [40]. A summary of the potential mechanisms involved in the pathogenesis of PABC is presented at Table 2. 

Due to its unique genetic and molecular profile, PABC demonstrates more aggressive biological behavior and distinct histological and immunohistochemical features. Several studies have shown that PABC seems to be more commonly associated with less favorable tumor characteristics, such as a reduced expression of estrogen (ER) and/or progesterone (PR) receptors, and of HER2/neu [27,41,42], although there are studies supporting that there are no significant differences in the expression of hormone receptors and HER2 between PABC and non-pregnancy-associated breast cancer [43]. PABC also demonstrates a marked propensity to become triple-negative breast carcinomas (TNBC), have an enhanced expression of potentially relevant cancer targets (e.g., PD1/PDL1, SRC, insulin growth factor and Wnt/β-catenin, RANK ligand), and have a low prevalence of tumor-infiltrating lymphocytes [44,45]. It is also worth mentioning that patients with PABC are at an increased risk of metastatic disease when compared to non-pregnant breast cancer patients. This difference can be explained by the aggressive biological and immunohistochemical profile of PABC, since a significant proportion of PABC patients are diagnosed with TNBC, a histological type linked to poor prognosis and high metastatic propensity. However, it is interesting that the metastatic patterns of PABC and non-pregnancy-associated breast cancer are similar, given that, in both cases, metastases are most commonly identified in the lungs, liver, brain, and in the skeletal system [26,44,46].

## 3. Maternal Adverse Outcomes

The combination of diagnostic delay and the restriction of treatment options during pregnancy is mainly responsible for the increased incidence of adverse outcomes that women with PABC may face, compared to breast cancer in general population, especially in terms of survival outcomes [47]. A delay of one month in diagnosis is equivalent to a 0.9% increase in the risk of metastatic nodal disease [48]. There is notable controversy regarding the impact of PABC on survival rates and prognosis, with conflicting results reported in the existing literature. Generally, the majority of PABC patients have advanced-stage disease with nodal involvement at the time of primary diagnosis. According to Johansson et al. [49], in a population of 317 patients with PABC, 55.5 and 12.6% of them were classified as of stage II and stage III, respectively, compared to 50.6 and 7.2% of non-PABC women with the same initial diagnosis (*p* < 0.001). Similar rates were reported by a retrospective multicenter clinical study on 164 women with PABC by Jin et al., in which stage I patients accounted for 9.1%, stage II accounted for 54.9%, stage III accounted for 24.4% and stage IV accounted for 2.4% of the study population [50]. Basaran et al. [47] studied a population of 20 PABC patients, 75% of whom had advanced-stage (III–IV) disease at the time of diagnosis. Previous studies have reported even higher rates; Ishida et al. [51] estimated that 65–90% of 192 PABC cases are primarily diagnosed with stage II-III disease, compared to 45–66% of patients with non-pregnancy-associated breast cancer. 

As expected, the survival outcomes of patients with PABC may vary, depending on the stage of the disease. The overall rate of maternal survival, when appropriate tailored treatment was applied, was estimated at 87.5% by Gilmandyar et al. [52], but it drastically decreases for stage IV patients, for whom the 5 year OS is 34% (95% confidence interval 21–46%) [53]. More specifically, in accordance with stage at primary diagnosis, survival rate declines as the disease stage increases, as follows: stage I, 100%; stage II, 86%; stage III, 86%; and stage IV, 0% [54]. In a retrospective study of 142 women with PABC, performed by Wang et al. [55], five-year overall survival (OS) and disease-free survival (DFS) rates were 76.8% and 63.5%, respectively, with T stage, N stage, and HER2 status, distinguished by univariate analysis, being significant prognostic factors for both OS and DFS. Bajpai et al. [56] further demonstrated that the 3 year event-free survival (EFS) was significantly different among PABC patients with early-stage, locally advanced and metastatic breast cancer, estimated at 82% (95% CI: 65.2–100), 56% (95% CI: 42–75.6%) and 24% (95% CI: 10.1–58.5%), respectively. 

In addition to the above, it is crucial to determine as to whether the prognosis is significantly different between women with PABC and women with non-pregnancy-associated breast cancer. Regarding existing evidence, there is discrepancy among the results of relevant studies that attempted to answer this research question. There is a plethora of studies supporting that survival outcomes are significantly poorer for patients with PABC, in terms of OS, DFS, progression-free survival (PFS), cause-specific survival (CSS) and relapse-free survival (RFS) [57,58,59,60,61,62]. Indicatively, Jo et al. [63] reported 5 year OS rates of 83.2% and 93.4% in patients with PABC and non-PABC patients, respectively (*p* < 0.001), as well as decreased 5 year DFS rates for women with PABC (72.2% vs. 83.8%, respectively). According to a retrospective multi-center study by Bonnier et al. [64], overall 5-year recurrence-free survival, metastasis-free survival and overall survival was lower, in a statistically significant manner, for patients with PABC compared to non-PABC patients, with multivariate analysis demonstrating that pregnancy was an independent and significant adverse prognostic factor. On the other hand, an increasing number of studies are pointing towards a different direction, by presenting poorer survival outcomes for women with PABC, which, however, do not differ in a statistically significant manner from the respective outcomes of non-PABC patients, especially after adjusting the comparing groups for known prognostic factors [15,65,66,67,68,69]. Amant et al. [68] has reported one of the largest cohort studies, which analyzed the prognosis of 311 pregnant women with PABC. After proper adjustments, the authors found no significant differences in DFS or OS between pregnant and nonpregnant patients with breast cancer, while multivariable analyses confirmed that pregnancy was not a factor that was associated with recurrence or death risk for the pregnant subpopulation. Another recent study by Li et al. [66] also demonstrated no significant difference between the EFS of patients with PABC and non-PABC patients with breast cancer (*p* = 0.655). Taking all the above into consideration, it is evident that existing data are therefore not consistent, and the level of evidence from the reported results is low, given that the majority of studies are retrospective. This controversy has created the need for a systematic combination of all existing evidence, in order to deduct safer conclusions. The first meta-analysis attempting to resolve this issue was performed by Azim et al. [16] in 2012, including 30 studies and a population of 3628 patients with PABC and 37100 non-pregnancy-associated breast cancer cases. The authors concluded that women with PABC had a significantly higher risk of death (pooled hazard ratio (pHR): 1.44; 95% CI [1.27–1.63]), while DFS analysis also resulted in a significantly higher risk of relapse associated with PABC (pHR: 1.60 [1.19–2.16]). A second, more recent meta-analysis by Shao et al. [70], performed in 2020 with 76 included studies, confirmed the results of the previous one by demonstrating that PABC was associated with poor prognosis for OS, DFS and CSS, and the pooled HRs with 95% CIs were 1.45 (1.30–1.63), 1.39 (1.25–1.54) and 1.40 (1.17–1.68), respectively. Conclusively, it can be deducted that PABC has a worse prognosis when compared to non-pregnancy-associated breast cancer.

Although decreased survival is undoubtedly the most severe adverse maternal outcome for patients with PABC, there are other complications that may greatly affect the overall health and the quality of life of these patients. Placental metastasis is extremely rare in women diagnosed with PABC during pregnancy, with only 17 cases described in the literature so far [71]. Despite the fact that PABC exhibits a particularly aggressive biological behavior, with an increased probability of distant metastases, the placenta is assumed to function as a non-supportive microenvironment for cancer cells [71]. Thus, placental metastases almost always occur in case of widespread hematogenous dissemination of the malignancy, and their detection is indicative of very poor maternal outcome [72]. Placental metastases may be underdiagnosed, due to their rarity, especially when the placenta appears macroscopically normal, as in such cases, histological examination of the placenta is frequently omitted. To avoid missing the diagnosis of placental metastasis, it is advised to request a detailed histopathological examination of the placenta by an experienced pathologist, in all patients diagnosed with PABC [71,73]. Usually, pathological report shows evidence of metastasis in the intervillous space, without suggestion of involvement of the fetal villous stroma or the fetal vascular circulation [74]. 

Finally, an important issue that can be often overlooked in women receiving treatment for PABC is the preservation of their reproductive health. A large proportion of patients with PABC will receive chemotherapy as part of their treatment plan, which may significantly affect their ovarian function and therefore their future fertility. Liao et al. [58] compared the effect of chemotherapy on ovarian function between 24 PABC patients and 48 non-PABC patients who had chemotherapy-induced amenorrhea. The resumption of menstruation, an indirect clinical sign of resumption of ovulation and ovarian endocrine function, was used to represent the resumption of ovarian function. The authors reported no significant difference in the rate of menstrual resumption between women with PABC and non-PABC patients at 6 months and 12 months after completing their chemotherapy regimen (*p* > 0.05). Moreover, an additional logistic regression failed to detect confounding factors that may have influenced this result. In addition to the above, another issue that may concern PABC survivors is the safety of future pregnancies, since the vast majority of patients with PABC are women of reproductive age, contrary to patients with non-pregnancy-associated breast cancer, who may wish to further expand their family after successful treatment of PABC. Approximately 50% of women with a history of breast cancer might desire a subsequent pregnancy, with only 4–7% of them becoming pregnant. Potential explanations for this low percentage are damaged fertility due to cancer treatment and fear of a negative impact of pregnancy on the development of breast cancer, which eventually discourages the patients from trying to get pregnant [50]. Several studies have been conducted to investigate the safety of pregnancy after breast cancer diagnosis and treatment, providing reassuring data showing that pregnancy after breast cancer diagnosis and treatment does not adversely affect survival [75,76,77]. Furthermore, there are three published meta-analyses suggesting that survival outcomes, mainly overall survival, are significantly improved among patients who became pregnant after breast cancer treatment than among those who did not, showing that pregnancy, especially when occurring at least two years after breast cancer diagnosis, does not jeopardize prognosis and could even contribute to more favorable survival rates [78,79,80]. In conclusion, existing evidence supports that women already diagnosed and treated for breast cancer can safely achieve future pregnancies, without compromising oncological safety. 

## 4. Obstetrical, Fetal and Neonatal Adverse Outcomes

### 4.1. Obstetrical and Fetal/Neonatal Complications Associated with PABC

The diagnosis and treatment of breast cancer during pregnancy may interfere with the progress of the pregnancy itself, potentially causing obstetrical complications that can directly or indirectly affect the viability and the health status of the fetus. Ethical aspects play a critical role in the management of PABC, since giving priority to not damaging the fetus significantly complicates any therapeutic approach in pregnancy, generally. A proportion of PABC patients may opt for a termination of pregnancy, when PABC is diagnosed during the first trimester, in order to avoid the inevitable restriction of treatment options [27]. However, there is no specific evidence to support that termination of pregnancy in the first or second trimester improves the maternal prognosis for patients with PABC [12,46,81]. It is entirely up to the expecting mother to decide as to whether to terminate or proceed with the pregnancy, a decision she should make after extensive counselling with the multidisciplinary medical team and according to her individualized needs [12]. The percentage of patients with PABC choosing to terminate their pregnancy is roughly estimated to reach up to 25% [82]. According to Zubor et al. (2018), in the case that pregnancy termination is decided by the patient, it can be performed either on the request of the patient up to the end of first trimester, or on the basis of oncological indication up to the end of the 24th week of pregnancy (the gestational age until which the termination of pregnancy for medical reasons is legally accepted may vary among different countries based on their legislation, with the 24th week of gestation being a common upper limit; clinicians should always adhere to the respective national legislation of the country in which they practice medicine) [12]. 

A plethora of obstetrical adverse outcomes related to PABC have been reported in a variety of studies, with prematurity identified as the most common one. Pregnancies complicated by breast cancer are at a greater risk of preterm delivery, as well as a preterm premature rupture of membranes (PPROM) [37,52]. These observations were confirmed by a large study including over 11 million births, with 772 PABC patients among them, which showed that pregnant women with PABC face a statistically significant risk of preterm delivery and PPROM [83]. Moreover, Esposito et al. demonstrated that PABC was positively associated with an increased incidence of labor induction or planned delivery (adjusted prevalence ratio—aPR = 1.80, 95% CI: 1.57–2.07) and Cesarean section (aPR = 1.78, 95% CI: 1.49–2.11) [84]. A higher prevalence of small-for-gestational-age embryos and stillbirth has also been noticed in pregnancies complicated by PABC [85]. Currently, there is limited information regarding the underlying reasons for the higher prevalence of preterm births and other obstetrical/fetal/neonatal adverse outcomes, in pregnancies complicated by breast cancer. It can be speculated that such complications may be a direct effect of the time and type of treatment itself (mostly chemotherapy), as described below. They may also stem from the cancer itself, but it is more probable that they are an iatrogenic effect of preterm induction of labor, in an effort to allow the use of more aggressive treatment protocols of the cancer postpartum; however, it is unclear if there is an association between time of diagnosis and the occurrence obstetrical/fetal complications [83].

PABC is also associated with an increased risk of early postnatal complications when compared to normal pregnancies, which are mainly caused by prematurity. Early neonatal complications include neonatal death, neonatal intensive care unit (NICU) admission, hematologic disturbances (especially neonatal anemia), low birth weight and other prematurity-related disorders (respiratory distress syndrome, metabolic disturbances, sepsis, infant jaundice, and necrotizing enterocolitis) [56,84,85,86], while low Apgar scores after birth are also more common in the neonates of PABC patients [84,87]. Finally, in addition to the aforementioned risk of placental metastasis from PABC, fetal metastasis can theoretically occur too, with extremely poor anticipated prognosis, however, there have been no reported cases of distant metastasis to the fetus. Fetal involvement would be possible when villous invasion of maternal cancer cells is observed, although the placental metastasis is usually limited to the intervillous space of the placenta. Placental epigenetic changes and subsequent potential long-term risks that infants of pregnancies with placental metastases may face should be further researched in future prospective studies [46,71,74,88].

### 4.2. Fetal/Neonatal Complications Associated with PABC Treatment

The administration of chemotherapy is considered safe and acceptable during the second and third trimesters of pregnancy for patients with PABC, but is contraindicated in the first trimester due to the high risk of fetal malformations. Before starting any oncological treatment, a fetal ultrasound should be performed, in order to eliminate the presence of pre-existing abnormalities that could be wrongfully attributed to the treatment [85]. In case of accidental administration of chemotherapy regimens during the first trimester, the risk of spontaneous miscarriage and congenital malformations is notably increased (14–19%), as fetal organogenesis occurring during this period can be severely disrupted by cytotoxic agents [10,87,89]. At the same time, a higher risk of pregnancy complications and fetal malformations cannot be completely excluded in the case of exposure to chemotherapy during the second and third trimesters, including fetal growth restriction, premature labor and PPROM, low birth weight, stillbirth and neonatal mortality (mostly as a result of prematurity) [10,37,85,90,91]. Transient hematotoxicity and fetal myelosuppression are rare, but potentially serious, neonatal complications caused by exposure to chemotherapy. In order to avoid this risk and subsequent hematological complications during the delivery, such as bleeding, sepsis, or death, at least a 3 week window between the last administration of chemotherapy and delivery is proposed, as to to allow fetal bone marrow to recover [85,87]. Fetal and neonatal cardiotoxicity secondary to intrauterine exposure to anthracyclines is a potential side effect of chemotherapy administered to PABC patients [11,84]. However, a recent study by Amant et al. [68] reported no harmful fetal cardiac effects after fetal exposure to doxorubicin during the second and third trimester. In general, regimens containing anthracyclines and taxanes are considered to be relatively safe for administration in the second and third trimester of pregnancy [11,85,92].

Fetal exposure to radiation can also cause adverse outcomes, which vary depending on gestational age and dose of radiation. Generally, a radiation dose below 0.1–0.2 Gy is considered safe for the fetus, but exposure to higher doses during the first two weeks after conception will probably lead to a failure of blastocyst implantation. Exposure to radiation 2–8 weeks after conception may cause fetal malformations in the developing organs, especially in the central nervous system, which is notably sensitive to radiation, with reductions in the intelligence quotient (IQ) and mental retardation being recognized as potential long-term adverse outcomes. The risk of radiation-associated fetal complications significantly decreases after week 25 of gestation, while appropriate shielding of the fetus during radiation treatment is required to reduce fetal exposure from scattered radiation. In general, radiation is not recommended as a treatment option for pregnant patients with PABC, and is reserved for patients with post-partum PABC [86,87].

Endocrine therapy is contraindicated for the treatment of breast cancer during pregnancy. Tamoxifen has a profound teratogenic effect, as it is positively associated with birth defects of the genitourinary tract, ambiguous genitalia, vaginal bleeding, craniofacial malformations, and fetal death/spontaneous abortion. Additionally, it increases the risk of breast cancer development in the offspring [3,89]. Regarding immunotherapy options, trastuzumab is also not recommended during pregnancy, as it affects fetal renal development when used during the second and third trimester of pregnancy, leading to reversible oligohydramnios or even anhydramnios. It is not known to cause fetal congenital malformations, but it may indirectly induce fetal asphyxia and death, secondary to the absence of amniotic fluid [10,37,85,89]. Finally, surgical treatment for PABC is safe throughout pregnancy, without significant risks for the fetus. Nevertheless, when indicated, sentinel lymph node biopsy should be performed with the use of Technetium-99m (99mTc) colloid solution. Blue dye and isosulfan blue are associated with an increased risk of anaphylactic maternal reaction and should be avoided, while methylene blue is contraindicated during the first trimester of pregnancy due to its teratogenic properties [37,89,90].

### 4.3. Long Term Neonatal Outcomes after PABC

The majority of existing evidence about long-term effects on neonates exposed in utero to treatment for PABC is derived from retrospective observational studies, and, thus, is of low quality. The results of studies focusing on identifying potential harmful long-term adverse outcomes after intrauterine exposure to chemotherapy are encouraging, showing no significant difference in attaining milestones, in neurocognitive behavior, in psychological, neurological development and educational performance, as well as in cardiac function, compared to children born from healthy women [3,10,37,86]. Moreover, no significant delays in puberty or cases of childhood cancer have been reported in children exposed to chemotherapy for breast cancer in utero [87,89]. However, some studies have shown an independent effect of prematurity on cognitive and neurodevelopmental outcomes, with the most immature infants facing the highest risk [68,93,94]. Longer follow-up data on cognitive, behavioral, and academic performances are necessary to support these findings. Regarding intrauterine exposure to potential teratogenic agents, tamoxifen elicits cellular alteration similar to diethylstilbestrol, which has been associated with the occurrence of adverse outcomes that can be more evident later in life. Although the use of tamoxifen is contraindicated in pregnant patients with PABC, accidental fetal exposure may produce long-term complications that can be revealed at an advanced age, apart from major malformations observed at birth [37,87,95]. A summary of reported adverse obstetrical, fetal, and neonatal outcomes associated with the diagnosis and treatment of PABC is presented at Table 3.

Future prospective research efforts dedicated to monitoring and systematically reporting short- and long-term complications are expected, in order to provide more reliable data, which will enable oncologists to improve the overall management of patients diagnosed with PABC during pregnancy.

## 5. Conclusions

Pregnancy-associated breast cancer is a clinical entity which should be distinguished from breast cancer occurring in the general population, due to its particularities in terms of epidemiology, pathophysiology, diagnostic and therapeutic management. The molecular and biological background of PABC is not yet fully elucidated, but its pathophysiological basis is traced to a combination of the hormonal and immune changes that occur during pregnancy, with breast involution and altered gene expression also significantly contributing to the pathogenesis of the disease. PABC exerts a particularly aggressive biological behavior, which is reflected in its distinct histopathological and immunohistochemical characteristics, and is associated with generally poor adverse maternal, obstetrical, fetal and neonatal outcomes. The majority of PABC patients have advanced-stage disease with nodal involvement at the time of initial diagnosis and face a significantly poorer prognosis, in terms of survival rates, compared to patients with non-pregnancy-associated breast cancer. Regarding obstetrical and fetal outcomes, the most common adverse events are preterm delivery and prematurity-associated neonatal morbidity. Short- and long-term neonatal treatment-associated complications might occur, even when safe and acceptable therapeutic options are administered during pregnancy. The systematic reporting of such adverse events in future longitudinal prospective studies will significantly contribute to the improvement of management of PABC patients, hopefully leading to a reduction in the incidence of unfavorable maternal and neonatal outcomes.

## Figures and Tables

**Table 1:** Specific characteristics of PABC—differences from non-pregnancy-associated breast cancer.

**Epidemiology**	Diagnosis at younger age (median age at diagnosis: 33 years) Increasing incidence as a result of a delay in childbearing
**Pathophysiology**	Pregnancy-associated changes in hormonal profile and immune response Breast involution after lactation Altered gene expression
**Histology &** **Immunohistochemistry**	Aggressive biological behavior Increased risk of metastatic disease Reduced expression of estrogen (ER) and/or progesterone (PR) receptors, and of HER2/neu Marked propensity to triple-negative breast carcinomas, high expression of PD1/PDL1, SRC, insulin growth factor and Wnt/β-catenin, RANK ligand, low prevalence of tumor-infiltrating lymphocytes
**Clinical** **manifestation**	Symptoms often perceived as physiological breast changes during pregnancy and lactation or benign lactation-associated irritations and diseases
**Diagnosis**	Delayed diagnosis due to late self-report of symptoms Advanced stage at diagnosis Limitations in the use of diagnostic modalities due to pregnancy (decreased sensitivity, potential harmful effects to the fetus)
**Treatment**	Restriction of treatment options due to potential teratogenic effects to the fetus (especially systemic therapies such as radiation, chemotherapy, hormonal therapy and immunotherapy) Substantially different designs of therapeutic strategies (time, dose and type of treatment adjusted to ensure fetal safety/viability)
**Prognosis**	Poor maternal survival outcomes

**Table 2:** Potential mechanisms involved in the pathogenesis of pregnancy-associated breast cancer.

**Changes in hormonal profile during pregnancy**	Enhanced breast cell proliferation and induction of onco-genetic procedures Increased growth of abnormal cells with malignant biological behavior
**Changes in immune response during pregnancy**	Cellular immunosuppression Increased immune tolerance/reduced immune control Enhanced inflammatory responses associated the with breast involution
**Breast involution after lactation**	Regression of fully differentiated functional mammary gland into pre-pregnancy condition Increased cell apoptosis, extended stromal remodeling, adipogenesis, and stimulation of inflammatory responses
**Altered gene expression**	Increased expression of genes involved in immune responses, cell cycle regulation, metabolism, aggressiveness, recurrence Altered expression of oncogenes, suppressor genes, apoptosis regulators, transcription regulators, genes associated with DNA repair mechanisms, and genes associated with cell proliferation and immune response Higher frequency of non-silent mutations, mutations in the mucin gene family, and germline mutations

**Table 3:** Adverse obstetrical, fetal, and neonatal outcomes associated with the diagnosis and treatment of PABC.

**Obstetrical** **adverse outcomes associated with PABC**	Termination of pregnancy, preterm delivery, preterm premature rupture of membranes (PPROM), induction of labor, birth via Caesarean section, small for gestational age fetus (SGA), stillbirth, placental metastasis
**Fetal/neonatal** **adverse outcomes** **associated with PABC**	Neonatal death, neonatal intensive care unit (NICU) admission, hematologic disturbances (especially neonatal anemia), low birth weight, other prematurity-related disorders (respiratory distress syndrome, metabolic disturbances, sepsis, infant jaundice, and necrotizing enterocolitis), low Apgar scores, fetal metastasis
**Fetal/neonatal** **adverse outcomes** **associated with** **treatment of PABC**	Chemotherapy (1st trimester): spontaneous miscarriage, congenital malformations Chemotherapy (2nd–3rd trimester): fetal growth restriction, premature labor, PPROM, low birth weight, stillbirth, neonatal mortality, transient hematotoxicity—etal myelosuppression Radiation: failure of blastocyst implantation, fetal malformations (especially in the central nervous system) Tamoxifen: teratogenic effects, birth defects of the genitourinary tract, ambiguous genitalia, vaginal bleeding, craniofacial malformations, fetal death/spontaneous abortion, risk of breast cancer development in the offspring Trastuzumab: oligohydramnios, anhydramnios Methylene blue (for sentinel lymph node biopsy): teratogenic effect
**Long-term neonatal adverse outcomes associated with treatment of PABC**	Indirect effect of prematurity on cognitive and neurodevelopmental outcomes

## Data Availability

Not applicable.
